# The clinical evaluation of a widefield lens to expand the field of view in optical coherence tomography (OCT-A)

**DOI:** 10.1038/s41598-024-57405-3

**Published:** 2024-03-23

**Authors:** Fritz Soecknick, Katharina Breher, Zahra Nafar, Sophie Kubach, Jochen Straub, Siegfried Wahl, Focke Ziemssen

**Affiliations:** 1 Department of Ophthalmology, University of Tübingen, Tübingen, Germany; 2 Institute for Ophthalmic Research, University of Tübingen, Tübingen, Germany; 3 Carl Zeiss Vision International GmbH, Aalen, Germany; 4 Carl Zeiss Meditec Inc., Dublin, CA USA; 5 Department of Ophthalmology, University of Leipzig, Leipzig, Germany

**Keywords:** Retinal diseases, Medical research, Applied optics

## Abstract

This study aimed to evaluate the clinical benefits of incorporating a widefield lens (WFL) in optical coherence tomography angiography (OCT-A) in patients with retinal vascular diseases in comparison to standard single-shot OCT-A scans. Sixty patients with retinal vascular diseases including diabetic retinopathy (DR) and retinal vein occlusion (RVO) were recruited. OCT-A imaging (PlexElite 9000) with and without WFL was performed in randomized order. The assessment included patient comfort, time, field of view (FoV), image quality and pathology detection. Statistical analysis included paired *t*-tests, Mann–Whitney *U*-tests and Bonferroni correction for multiple tests, with inter-grader agreement using the kappa coefficient. Using a WFL did not lead to statistically significant differences in DR and RVO group test times. Patient comfort remained high, with similar responses for WFL and non-WFL measurements. The WFL notably expanded the scan field (1.6× FoV increase), enhancing peripheral retinal visibility. However, image quality varied due to pathology and eye dominance, affecting the detection of peripheral issues in RVO and DR cases. The use of a WFL widens the scan field, aiding vascular retinal disease imaging with minor effects on comfort, time, and image quality. Further enhancements are needed for broader view angles, enabling improved quantification of non-perfused areas and more reliable peripheral proliferation detection.

## Introduction

Imaging of the retina is an elementary component of diagnostics in ophthalmology. Special attention is paid to the vascular system of the retina, where blood vessels can be viewed and assessed directly. Retinal vasculature also serves as the reflection of the bodily vessel status in systemic cardiovascular diseases^1–3^. Ocular vasculature can be observed via ophthalmoscopy and optical imaging, such as fundus photography, fluorescence angiography and optical coherence tomography angiography (OCT-A)^4,5^. OCT-A as the most novel angiography imaging tool is based on optical coherence tomography and therefore provides cross-sectional imaging of tissue in combination with the automated detection of vessels and capillaries in all retinal plexus. The basic functionality of OCT-A technology is to determine the positions of erythrocytes during the passage of a vessel via speckle noise and to distinguish blood vessels from surrounding static tissue. Consequently, a three-dimensional construction of perfused vessels of the retina and choroid can be made and vascular parameters, such as vascular density, perfusion density, area of non-perfusion and foveal avascular zone description, can be derived^6–8^. OCT-A holds several advantages in comparison to the standard methods for vascular assessment. In contrast to fundus imaging, OCT-A provides perfusion information for various retinal and choroidal plexus in addition to the small capillaries. When compared to fluorescence angiography, OCT-A presents a non-invasive and faster procedure without the need for a contrast agent.

OCT-A has already been proven to be beneficial during the diagnosis, monitoring and treatment process of vascular diseases of the eye, like diabetic retinopathy or retinal vein occlusion^9–13^. However, the presence of angiographic abnormalities in both disease groups is not limited to the central region alone. For example, in diabetic retinopathy (DR), between 5 and 15% of patients showed peripheral lesions outside of the standard 7-field fundus imaging protocol, which leads to a more severe grading of retinopathy^14,15^. Furthermore, peripheral ischemia is associated with a higher risk for future disease progression^16,17^. In retinal vein occlusion (RVO) for example, the extent of non-perfused retinal areas plays an important role in the clinical prognosis, like neovascularization development, and treatment response^18–20^. This also transfers to OCT-A where a wider scan field for the evaluation of the periphery offers a clinical benefit.

The maximum size of a single shot scan of modern OCT-A devices is currently in the range of approximately 15 × 15 mm (corresponding to approximately 52° × 52°) at the posterior pole of the retina. Alternative possibilities, such as the montage function of several single images in different fixation directions are accompanied by several disadvantages: distortions and discontinuities after image stitching, prolonged examination time and unstable fixation changes of the patient. To avoid the above disadvantages of the montage function, but to maintain simultaneous imaging of the retinal periphery, a widefield lens (WFL) was developed. The WFL enables the optical enlargement of the scan area in the single image by a factor of 1.7—from 52° to 90°^21,22^. Since a WFL significantly reduces the distance, effects on the resolution (unchanged format of the stored image with a larger angle) as well as the stability of the eye tracking mechanism could occur, we saw the need to systematically and prospectively investigate the impacts of its use. In OCT-A imaging, artifacts often complicate interpretation and image analysis^23,24^, especially when segmentation is difficult with increasing curvature toward the periphery or opacities of the optical media (lens and vitreous) further reduce signal strength^25,26^. Due to the novelty of the WFL, the study aimed to evaluate its usefulness, benefits and limitations in clinical practice in comparison to a standard single-shot OCT-A scan in patients with proliferative diabetic retinopathy and retinal vein occlusion. The assessment of the unauthorized medical device required the prior consent of the patients according to a test plan reviewed by the ethics committee.

## Materials and methods

### Study participants

This study is a cross-sectional observation cohort study. Study participants were consecutively recruited from the outpatient clinic of the University Eye Hospital Tübingen. As this research involves human research participants it follows the Declaration of Helsinki and data protection regulations. After clarification of the patients’ voluntary interest to participate in the study and after an explanation of the study, written informed consent was obtained from all subjects and/or their legal guardians. The Ethics Committee of the Faculty of Medicine of the University of Tübingen approved this study. Inclusion criteria were a confirmed diagnosis of RVO or the presence of DR in at least one eye. Only patients with inadequate capacity to consent, present epilepsy or very severe tremor were excluded. Further exclusion criteria were high spherical (> ± 8 D) and cylindrical (> ± 4 D) refractive errors. The patient was seated in front of the examination device in a calm posture while following the instructions of the examiner in a focused manner. The ability to read and understand simple sentences in German was a prerequisite for understanding the examiner’s instructions and answering the questionnaire. In total, the RVO and DR group both consisted of each 30 participants with equal distribution between the gender (RVO: 16/14, DR: 15/15, male/female). The mean age of subjects in the RVO and DR group was 68 ± 8 years and 64 ± 12 years, respectively.

### Examination devices

**Figure 1:**
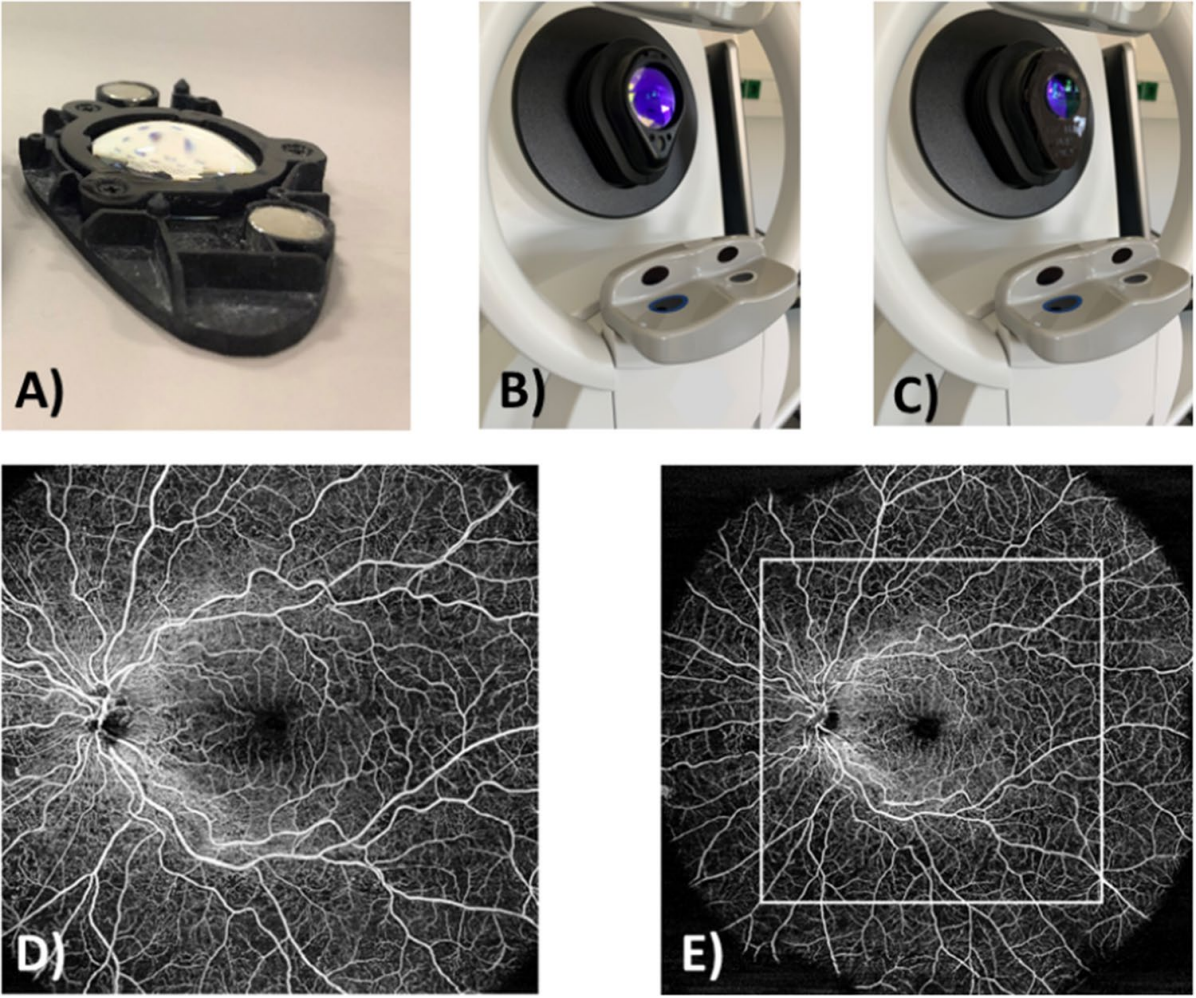
(**A**) The WFL (widefield lens) in the black plastic housing with magnets; (**B**) The OCT ocular without the WFL; (**C**) The OCT ocular with the WFL; (**D**) OCT-A enface image without WFL; (**E**) OCT-A enface image with WFL and comparison to the original FoV without WFL.

The PlexElite 9000 OCT device (Carl Zeiss Meditec, Inc., Dublin CA, USA) with software version 2.1 was used for OCT-A imaging. All OCT-A images were acquired at a scanning frequency of 200 kHz. The device has a central wavelength between 1040 and 1060 nm and has a sweep range from 980 to 1120 nm. For ultra-widefield imaging, an additional prototype WFL was used. The lens is embedded into a black plastic housing measuring approximately 9 cm × 5 cm that can be mounted on the ocular of the OCT devices via magnets (see Fig. 1A–C). Axial length was determined based on laser interference biometry using the IOL-Master 700 (Carl Zeiss Meditec AG, Jena, Germany). The axial length of the examined eye, which is individual for each patient, is needed to determine the actual field of view (FoV) of the scan on the retina. An ultra-wide angle photograph was taken of both eyes for reference (Zeiss Clarus 700, Carl Zeiss Meditec AG, Jena, Germany).

### Study protocol

The entire examination procedure was divided into multiple stations. Firstly, the OCT-A images were acquired with and without WFL in randomized order (previously generated randomization list). If both eyes presented with pathologies, the OCT-A measurements were performed on the better-seeing eye first. The participants positioned themselves in a comfortable seated posture in front of the device with propping the chin and forehead against the support. The pupil was then centered by the examiner and the image focus was adjusted to compensate for participants’ refractive errors. In the case of scanning without WFL, the scan process was then started. In the case of scanning with WFL, the patient was moved a few centimeters away from the device after the setup and the lens was attached to the eyepiece of the OCT-A. The patient then resumed the same position as well as possible to achieve the same position on the device. The focus then was re-shifted by about − 13 D to compensate for the WFL lens power and to obtain a clear fundus image again. Afterward, the scanning process could be started. After each scan with and without WFL, the participant was asked to complete a questionnaire for subjective impressions regarding time duration and comfort. At last, ocular biometry including axial length and keratometry values was performed.

### Analyzed parameters

#### Time duration

The duration of all individual OCT-A acquisitions with versus without WFL was determined to the second using the stopwatch function of a smartphone (iPhone XS; Apple Inc., Cupertino CA, USA). To obtain comparable results, the duration was measured consistently from the time of patient positioning in the OCT-A device until the time when the software indicated the acquisition as completed.

#### Questionnaire

In addition to the objective parameters, the subjective rating of the volunteers was also collected through two identical questionnaires. The participant received a questionnaire after the first examination (without or with the WFL, depending on the randomization group) and the same questionnaire after the second examination and evaluated the procedure. The questions were as follows:

1. How would you rate this measurement procedure overall?
2. How comfortable was the positioning and alignment of the device for you?
3. Do you consider the duration of the entire measurement procedure to be reasonable?

Continuous scales were used to answer three questions, where the participants indicated their ratings via a freehand marking on a line of 10 cm length, corresponding to a scale between 0 (worst rating) and 10 (best rating). The exact rating was then read as the distance in mm from the 0-marking using a ruler.

#### Field of view (FoV)

The calculation of the imaged retinal area was performed with the computer software Matlab R2021b (MathWorks, Natick, MA, USA). In the exported enface images, the outer boundaries of the imaged area were manually defined. From the given parameters axial length and acquisition angle with and without WFL, the imaged area was then calculated in pixels and square millimeters and compared. An example of the enlarged FoV of an OCT-A enface image with WFL compared to an OCT-A enface image without WFL is shown in Fig. 1D,E.

#### Image quality

Two experienced ophthalmology specialists, masked to the clinical information and WFL use, independently graded the image quality. The images were provided in randomized order. To ensure a complete blinding regarding the recording method, all OCT-A scans, both those acquired with and without a WFL, were cropped to an identical size on Matlab R2020a. This was done to avoid the possibility that altered margins resulting from the WFL could be used to identify the use. Afterward, the image quality for the following criteria was graded: “vascular quality,” “motion artifacts,” and “shadows/reflections/artifacts.” To ensure consistent quality criteria within the grading system, the grade points for classification were defined by giving specific example images. The evaluation in the scoring system was from 1 (worst rating) to 5 (best rating).

#### Existence of peripheral pathologies

The existence of peripheral pathologies was evaluated on scans with WFL by two ophthalmologist graders. For this purpose, the evaluators were shown the peripheral image sections in the enface images with WFL, which were only visible with WFL, while the central 15 × 15 mm image section was cropped out using Matlab R2020a. The graders had to indicate whether they could make a sure diagnosis by only using the peripheral part of the OCT-A image. Moreover, they estimated the respective image quality on a scale from 1 to 5.

#### Diagnosis of pathological signs

First, independent ophthalmology specialists made the findings of the present disease stage. For the OCT-A images with and without the WFL of the subject group “RVO”, the extent of the non-perfused areas (NPA) in disc diameters was estimated. In the patient group “DR”, the size of NPAs was also assessed, as well as the size of neovascularizations elsewhere (NVE) and at the optic disc (NVD).

### Statistical analysis

Data preparation and graphical presentation were performed using Microsoft Excel and Microsoft PowerPoint v. 16.62 (Microsoft Corporation, Redmond, WA, USA). Statistical analysis of the data was performed using the computer software IBM SPSS Statistics v.27 (International Business Machine Corporation, Armonk, NY, USA). All data were tested for normal distribution using the Shapiro–Wilk test. For the analysis within the RVO or DR group, a paired *t*-test (parametric data) or Wilcoxon paired comparison test (non-parametric data) was used. For cross-group comparisons, the unpaired *t*-test or Mann–Whitney U-test was applied. If multiple tests had to be performed, the underlying alpha and significance level of 0.05 was adjusted by Bonferroni correction. For inter-grader agreement, the kappa coefficient was calculated.

## Results

### Time duration

The recording time was evaluated both within the subject groups RVO and DR with and without WFL and between pathology groups, see Table 1. While the duration of measurement with the WFL was slightly longer for both pathology groups (between 85 and 123 s) than without WFL (57–88 s), there was no statistically significant difference between both modalities and the pathology group (all p > 0.05 adjusted).

**Table 1:** Complete chair times (s) for the different pathology groups with and without WFL, given as median and IQR. No statistically significant differences were found (all p > 0.05 adjusted).

	Without WFL	With WFL
RVO		
Better eye	88 [61–110]	123 [87–200]
Worse eye	69 [50–88]	96 [77–118]
DR		
Better eye	88 [54–110]	100 [83–131]
Worse eye	57 [32–76]	85 [66–120]

### Questionnaire

Subjective patient rating is presented in Table 2. All ratings were consistently high with median ratings ≥ 8.4 with WFL and ≥ 8.8 without WFL. For all questions and both pathology groups, there was no statistically significant difference for measurements without vs. with WFL (all p > 0.05 adjusted).

**Table 2:** Questionnaire ratings with and without WFL, given as median and IQR. No statistically significant differences were found (all p > 0.05 adjusted).

	Without WFL	With WFL
RVO		
Overall rating	8.7 [7.7–9.2]	8.5 [6.8–9.2]
Comfort	8.9 [8.1–9.5]	8.4 [6.2–9.2]
Time duration	9.2 [8.5–9.7]	9.2 [8.6–9.8]
DR		
Overall rating	9.0 [7.9–9.7]	8.9 [7.4–9.7]
Comfort	8.8 [7.8–9.5]	8.8 [7.7–9.5]
Time duration	9.4 [8.5–9.7]	9.3 [8.8–9.7]

### Field of view (FoV)

**Figure 2:**
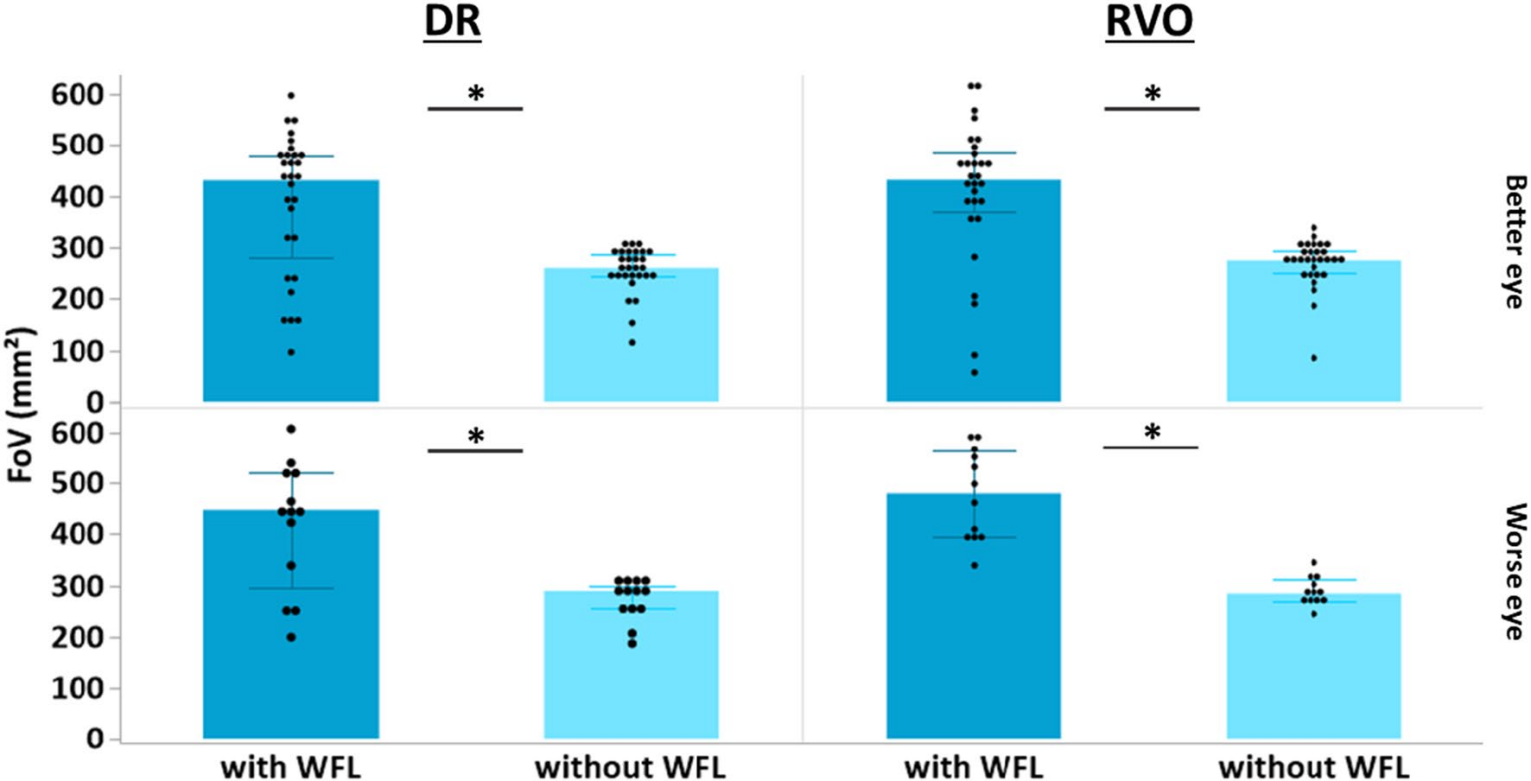
Comparison of the actual FoV without and with WFL for the DR and RVO groups with the better and worse eye, respectively (*p ≤ 0.05).

A graphical representation of the scan field size in mm^2^ for the different scan protocols and patient groups can be seen in Fig. 2. There was a significant increase in scan field size in subject group RVO in both eyes (better eye with WFL 431 mm^2^ [IQR 384–483 mm^2^] vs. without WFL 274 mm^2^ [IQR 250–286 mm^2^], p < 0.001; worse eye with WFL 480 mm^2^ [IQR 398–592 mm^2^] vs. without WFL 284 mm^2^ [IQR 272–302 mm^2^], p = 0.001). The DR group also showed a significant increase in scan field size at both eyes (better eye with WFL 431 mm^2^ [IQR 315–478 mm^2^] vs. without WFL 260 mm^2^ [IQR 245–285 mm^2^], p < 0.001; worse eye with WFL 447 mm^2^ [IQR 339–518 mm^2^] vs. without WFL 289 mm^2^ [IQR 254–296 mm^2^], p = 0.004). There was no significant difference in scan field enlargement with WFL between subject groups RVO and DR at the better eye or worse eye (all p > 0.05).

### Image quality

**Figure 3:**
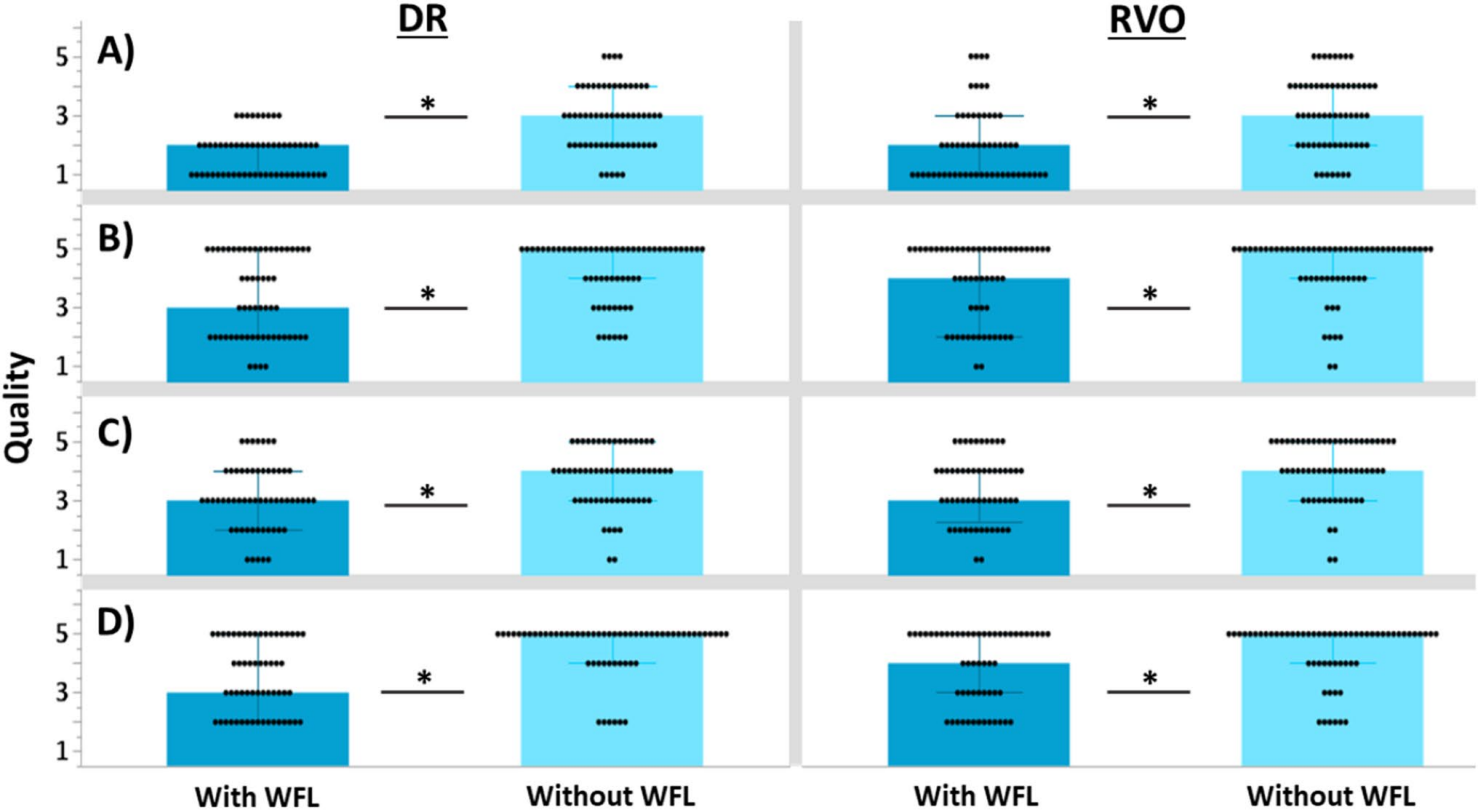
Averaged image quality grading from two ophthalmologists of the better eye for Row (**A**) the foveal avascular zone, Row (**B**) motion artefacts, Row (**C**) shadowing and Row (**D**) vascular quality (*p ≤ 0.05).

For the subject group RVO, the intergrader agreement was κ = 0.94, and for the group DR, κ = 0.93. The values do not suggest arbitrary scoring by either grader and indicate excellent reliability of the scores. In both pathology groups, the better eye showed a significant difference in the evaluation of FAZ quality (RVO: p = 0.003; DR: p < 0.001, see Fig. 3), whereas there was no significant difference in the worse eye (both groups p > 0.05). The same tendency was observed for the other image parameters like vascular quality (Fig. 3), motion artifacts (Fig. 3) and shadowing (Fig. 3). In subject group RVO, there was a significant reduction in the quality of presentation for all three categories at the better eye using the WFL compared to the standard OCT-A acquisition (vascular quality: p = 0.037; motion artifacts: p = 0.030 and shadowing: p = 0.002), while the presentation at the worse eye was not significantly different in any of the three categories (all p > 0.05). Similarly, in the DR pathology group, image quality with WFL was reduced (vascular quality: p = 0.001; motion artifacts: p = 0.004; shadowing: p = 0.021) and at the worse eye for the assessment of "shadow" (p = 0.014) while the presentations at the worse eye did not differ significantly in the other two categories (p > 0.05).

### Existence of peripheral pathologies

**Figure 4:**
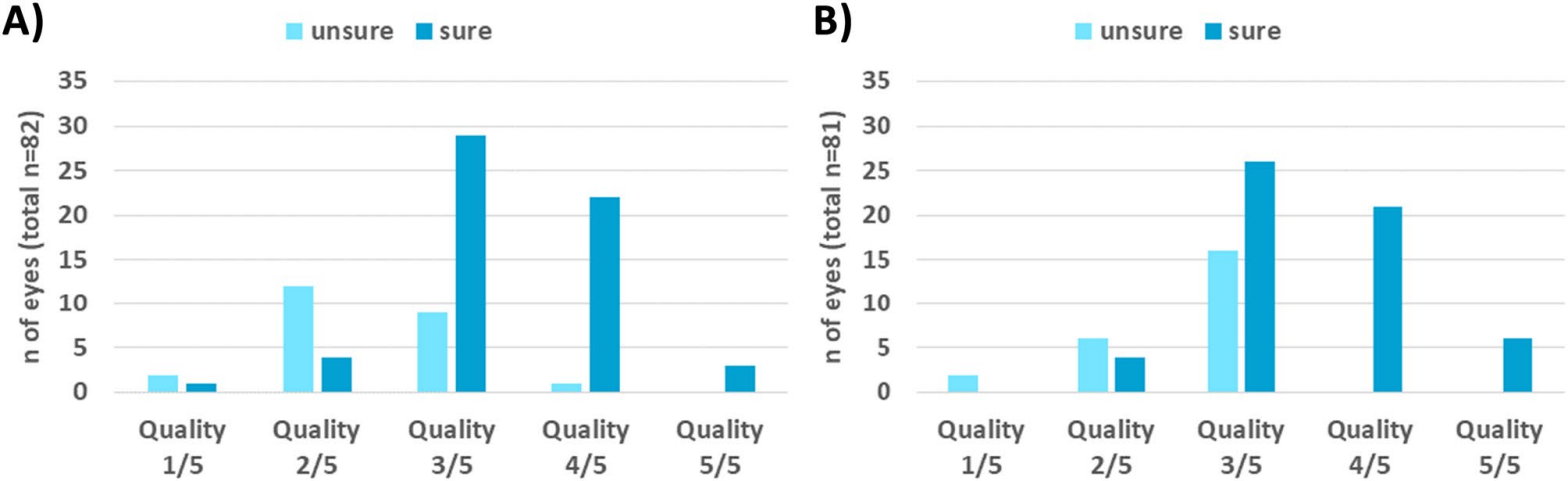
Number of eyes where pathologies could be surely excluded/confirmed or not, when only the peripheral area visible with the WFL was presented for (**A**) DR and (**B**) RVO.

The frequency of a sure confirmation/exclusion of pathology for the DR and RVO group is displayed in Fig. 4, depending on the quality rating of the image snippet. In the quality categories 1/5 and 2/5, 26% (DR) and 33% (RVO) of images could already be used meaningfully to confirm or exclude pathological signs. In the quality category 3/5, the cumulative number rose to 76% and 62% for the DR and RV group, respectively. Quality categories 4/5 and 4/5 allowed a cumulative confirmation or exclusion of pathological signs by 96% (DR) and 100% (RVO).

### Diagnosis of pathological signs

As seen in Table 3, there was no significant difference in NPA size between both lens modalities in the RVO group (better eye p = 0.067; worse eye p = 0.257). There was neither a significant difference in NPA size between the WFL and non-WFL group in DR patients (better eye p = 0.280; worse eye p = 0.828). Regarding NVE/NVD detection in the DR group, a statistically significant difference could be found in the better eye (p = 0.005), but not in the worse eye (p = 0.906).

**Table 3:** Estimation of NPA and NVD/NVE in images with and without WFL, given as median and IQR (*p ≤ 0.05 between both lens modalities).

	Without WFL	With WFL
RVO		
Better eye: NPA	2 [1–3]	2 [1–4]
Worse eye: NPA	8 [2–13]	6 [4–15]
DR		
Better eye: NPA	7 [3–14]	5 [3–11]
Better eye: NVD/NVE*	0 [0–3]	0 [0–1]
Worse eye: NPA	5 [4–7]	4 [3–12]
Worse eye: NVD/NVE	0 [0–2]	0 [0–1]

## Discussion

The study aimed to evaluate the clinical value of a WFL as an attachment to an OCT-A device in retinal vascular pathologies. The focus of the current study was laid on the image quality, the size of the scan field and the resulting consequences in the interpretation of retinal perfusion or disease-relevant parameters. It could be shown that the WFL achieved a significant increase of the FoV in combination with a facilitated clinical diagnosis. At the same time, the examination time was not significantly prolonged using the WFL and the subjective well-being of the patient during the examination was not negatively influenced by the WFL. This indicates a good clinical usability of the WFL.

A short examination time is advantageous for both the examiner and the patient. The OCT-A examination is usually performed in the outpatient department of an ophthalmic clinic and requires constant presence by trained medical staff. Framme et al. performed a study to calculate the costs and services for an ophthalmology outpatient clinic. Here, personnel costs represented close to 80% of total costs^27^. In the present study, no significant increase in examination time with WFL was found. Therefore, the possible diagnostic added value of the WFL is achieved without a significant additional personnel effort and costs. Moreover, the examination time influences the patient's attention during the examination procedure. A longer examination time raises the risks for fixation instability and decreased image quality. The relation between examination time and attention of the subject was also reported by Wylęgała et al. in the example of OCT-A examination of children and patients of higher age and they describe this correlation as one of the greatest causes of limitation in OCT-A diagnostics^28^. Besides, OCT-A diagnostics presents an alternative imaging method to fluorescence angiography, where an important advantage is the shorter examination time of OCT-A^29^. Even with WFL, OCT-A can significantly limit the time factor compared to the more time-intensive fluorescence angiography. Even in contrast to montage OCT-A from multiple individual scans, which Amato et al. describe as time- and labor-intensive, widefield imaging with WFL could be more time efficient^30^.

The enlargement of the scan field of an OCT-A image to 90° FoV represented the core of the current study. As a standard of current OCT-A technology, maximum scan sizes of about 12 × 12 to 15 × 15 mm^2^ are feasible, corresponding to a FoV of up to 55°^31^. However, the periphery outside of 55° is an important area of the retina for the diagnosis of retinal vascular pathologies. Attiku et al. demonstrated in a study that ultra-widefield imaging rarely changes the pre-determined severity of DR but does reveal new vision-impairing lesions outside the ETDRS-7 field and that widefield imaging is particularly important for DR^15^. This finding is consistent with previous study results, which indicate that additional retinal lesions in the periphery can be visualized by WFL^21^. Another study suggested that the presence of predominantly peripheral lesions is associated with a risk of progression regardless of the stage of DR^32^. This finding is true not only for patients already suffering from DR, but possibly also for patients with diabetes mellitus without clinical retinopathy^33^. This could result in an even earlier diagnosis with more satisfactory results in treatment and prognosis. The current study showed that albeit there is a statistical, but no clinical difference in the detection of NVE/NVDs with and without WFL in DR patients. Moreover, it revealed that the non-perfused areas even tend to be slightly underestimated with the WFL. Both results seem counterintuitive at first. However, the reduction in quality due to the clinical picture of DR^34^ in combination with the need for high resolution for NVE, NVD and NPA on the capillary level in DR can explain this outcome. Since this is less the case with RVO in comparison due to its physiological nature, the present results are conclusive in this regard. In retinal vein occlusion, the extent of non-perfused retinal areas plays a key role in the clinical prognosis, like neovascularization development, and treatment response as well^18–20^. This also transfers to OCT-A where a wider scan field for the evaluation of the periphery offers a clinical benefit. The relevance of the periphery was also demonstrated in myopia, where peripheral retinal and choroidal changes correlated with the severity of myopia^35^.

The presented work has the advantage of allowing objective comparisons of the FoV, corresponding to precise image angles. Certainly, the covered areas are still far from the information content of today’s ultra-wide angle angiographies^17^. Nevertheless, the posterior pole with the assessment of central ischemia and midperipheral localization should be of importance for vascular diseases. Bigger is better, even if the unmet list of desires is the reason that invasive angiography still retains a clinical value (Supplementary Information).

OCT-A images require sufficient imaging quality for diagnostic usability. Inadequate imaging quality, whether due to the procedure itself or to exogenous factors, can only contribute to a limited extent to a valid diagnosis. This was also shown by the current study: On the one hand, in images of lower quality, graders are only sure of their findings in one-third and one-fourth of the cases of RVO and DR, respectively. On the other hand, it was observed that the presence of pathology can be reliably excluded or confirmed with sufficiently good quality^21^. Regardless of the occurrence of quality-reducing artifacts or the like, it must be noted that the resolution of the images is significantly reduced by switching on the converging lens, as more area is displayed with the same number of pixels. This could have a negative impact on fine image details like capillaries.

Patient comfort is an evaluation parameter that has not yet been considered previously when investigating widefield OCT-A imaging. Even with WFL, OCT-A examination remains a non-invasive procedure, but the use of the additionally inserted lens during the measurement procedure could still influence the patient acceptance. The results of this study show that WFL does not significantly affect the patients’ comfort levels. Acceptance for the examination through high comfort could have both short- and long-term effects concerning a patient’s treatment course. Firstly, willingness to cooperate is increased, which might have a positive effect on imaging quality. On the other hand, if OCT-A is used as a regular follow-up examination, a high acceptance for the examination could ensure that follow-up appointments are kept in the long term and sustainably.

Important study limitations must be considered. While the cohort represented an everyday-relevant group—despite the possible pretreatment with intravitreal drugs, the measurements were performed with only one type of device. For other manufacturers, the use of put-in-front loupes might fail because of the mechanics or optical path distance. Therefore, no derivations or comparisons with individual devices, which already claim larger sizes or areas for themselves, are possible. Grading followed the usual specifications of a certified reading center, but the clinical relevance of the readings could not be/was not assessed later by this cross-sectional evaluation.

## Conclusion

The study found that the WFL shows good clinical usability by significantly enlarging the scan field of a single-shot OCT-A scan. This has positive effects on the diagnosis of vascular retinal diseases like DR and RVO, while has no effect on patient comfort and only slightly decreases image quality. Further studies are needed to prove the usability beyond DR and RVO, and in particular to evaluate its effect on treatment decisions. Although the potential magnification provides hope, the largest possible FoV remains desirable, which could resolve neovascularization and vascular processes up to 180° or 200° similarly to ultra-widefield imaging^4^.

### Supplementary Information

Supplementary Information.

## Data Availability

The datasets generated during and/or analyzed during the current study are available from the corresponding author upon reasonable request.
