# Supplementary material for: The clinical evaluation of a widefield lens to expand the field of view in optical coherence tomography (OCT-A)
Source: Sci Rep. 2024 Mar 23;14:6936. doi: 10.1038/s41598-024-57405-3 (PMC10960788; doi:10.1038/s41598-024-57405-3)

Supplementary material; Submission ID cd95165b-5452-49bf-a63b-25d2f8ecf29e

Comparison of the quality differences in the characteristics considered of the same eye

FAZ

with WFL, good quality (4/5)

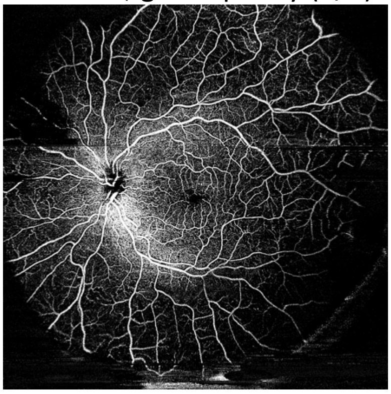

without WFL, good quality (5/5)

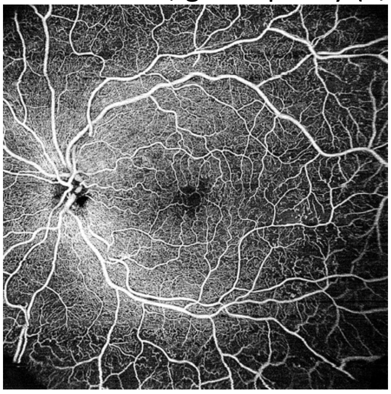

with WFL, bad quality (1/5)

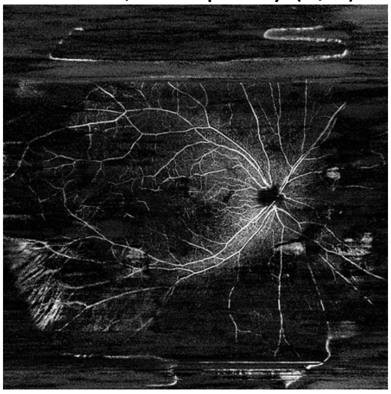

without WFL, good quality (5/5)

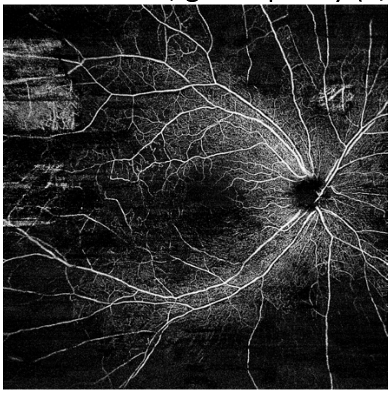

Motion artefacts

with WFL, good quality (4/5)

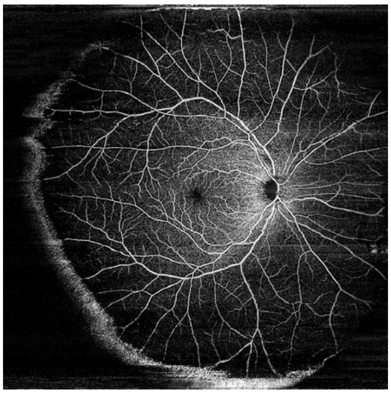

without WFL, good quality (5/5)

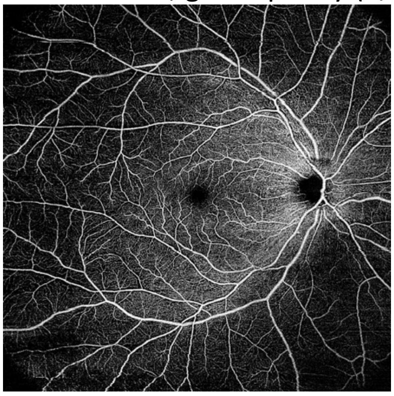

with WFL, bad quality (1/5)

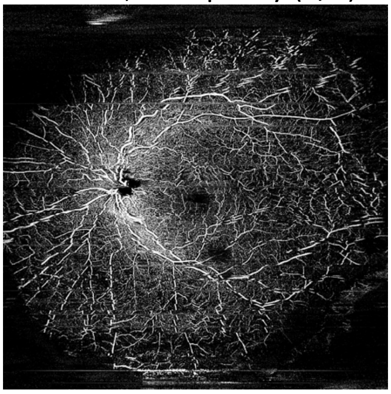

without WFL, good quality (5/5)

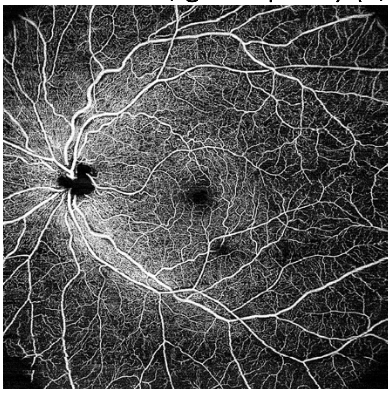

Shadowing

with WFL, good quality (5/5)

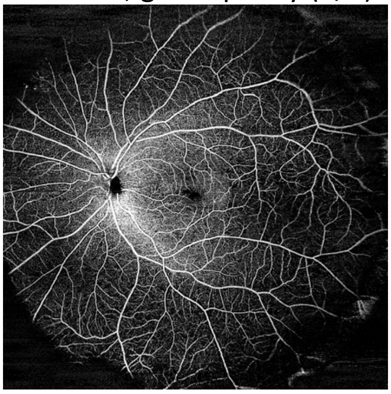

without WFL, good quality (5/5)

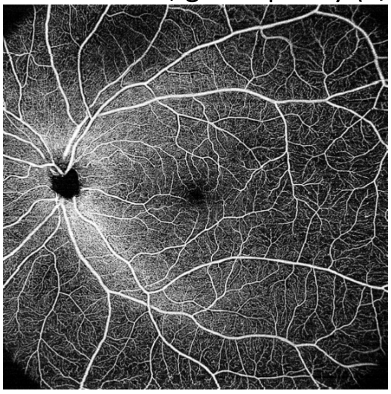

with WFL, bad quality (2/5)

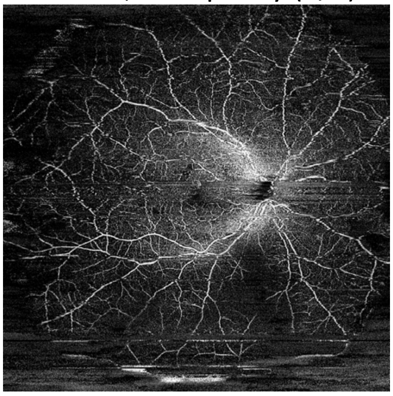

without WFL, good quality (4/5)

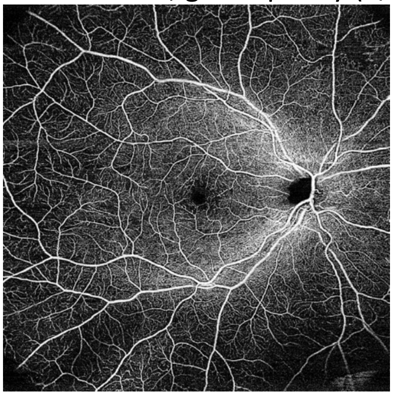

Vascular quality

with WFL, good quality (5/5)

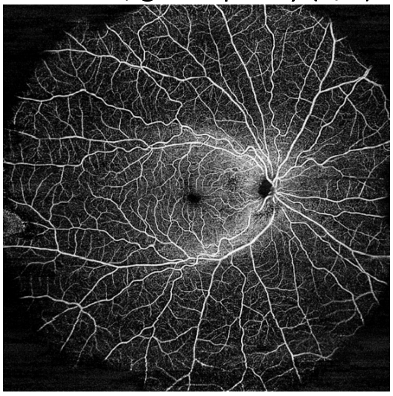

without WFL, good quality (5/5)

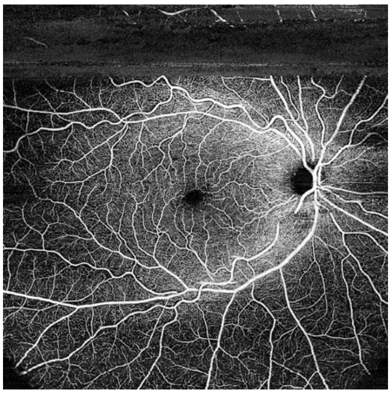

with WFL, bad quality (2/5)

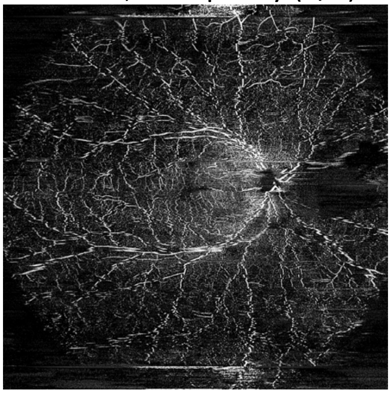

without WFL, good quality (5/5)

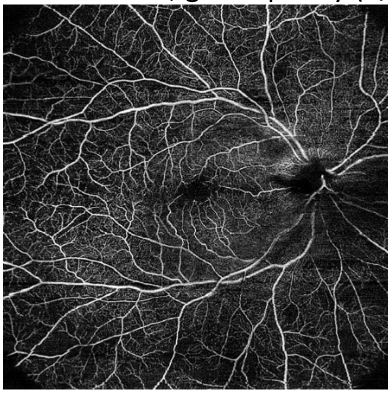

Supplement: Supplementary file 1 — Supplementary Information. [file 41598_2024_57405_MOESM1_ESM.pdf]
